# Structure and diversity of intestinal methanogens in black carp (*Mylopharyngodon piceus*), grass carp (*Ctenopharyngodon idella*) and water samples

**DOI:** 10.1371/journal.pone.0316456

**Published:** 2025-02-13

**Authors:** Chengxing Long, Peiyang Wang, Jieqi Wu, Jialin Liu, Zhoujin Tan, Wenge Li

**Affiliations:** 1 Science and Technology, Hunan University of Humanities, Loudi, Hunan, China; 2 Loudi Fisheries Science Research Institute, Loudi, Hunan, China; 3 Hunan University of Chinese Medicine, Changsha, Hunan, China; 4 Hunan Institute of Nuclear Agricultural Science and Space Breeding, Changsha, Hunan, China; Universiti Malaysia Kelantan, MALAYSIA

## Abstract

The present research investigation aims to examine the community features of methanogens in the intestinal tract of black and grass carp, as well as their association with methanogens in water samples. Samples of black carp, grass carp and water in a pond were gathered in Spring 2021. Using the Illumina HiSeq 2500 high-throughput sequencing platform, the metagenomic mcrA gene sequences of black carp, grass carp and cultured water specimens were determined and analyzed. The outcomes indicate that the richness and diversity of methanogens in the intestinal tract of black and carp grass carp were highly correlated with the cultured water. Five bacterial genera were found in the three sets of samples, *Methanosarcina*, *Methanocorpusculum*, *Methanospirillum*, *Methanobacterium* and *Methanofollis*, in which *Methanosarcina* and *Methanocorpusculum* were the dominant genera. In addition, *Methanosarcina* had the greatest amount in grass carp and *Methanocorpusculum* had the greatest quantity in black carp. In conclusion, *Methanosarcina* and *Methanocorpusculum* were the main methanogens in the digestive tract of black and grass carp and culture water, and hydrolytic fermentative bacteria were its main metabolic substrate, hydrotrophic was its main metabolic pathway. The results will provide a reference for the relationship between intestinal methanogens and aquaculture and the greenhouse effect.

## Introduction

*Methanogens*, also referred to as Methanogenic archaea, constitute a class of archaeal microorganisms capable of generating methane gas within an obligate anaerobic environment. They utilize substrates such as hydrogen, carbon dioxide, acetic acid, formic acid, methanol, and methylamine, among others, demonstrating a widespread distribution [1–4]. Methanogens are abundant and diverse. According to the Handbook of Berger Bacteria Identification (ninth edition), as of 2016, methanogens has developed into 4 classes (*Methanobacteria*, *Methanococci*, *Methanomicrobia*, *Methanopyri*) and 5 orders (*Methanococcales*, *Methanosarcinales*, *Methanopyrales*, *Methanomicrobiales* and *Methanobacteriales*) [5]. Methanogens mainly generate methane through the H_2_/CO_2_ reduction pathway and acetic acid fermentation pathway, while the methyl trophication pathway mainly occurs in specific environments such as river and pond sediments [6, 7].

Methane (CH_4_) is a greenhouse gas produced by methanogens decomposing organic matter under anaerobic conditions, and its contribution to global climate warming is second only to that of carbon dioxide (CO_2_) [8, 9]. Many CH_4_ accumulated on the earth come from the action of microorganisms. Because methanogens play a vital role in the natural carbon cycle, and CH_4_ is the 2^nd^ largest greenhouse gas contributes to global warming, methanogens and the mechanism of methane production have attracted much attention from researchers [10]. Especially in recent years, researchers have conducted in-depth discussions on the living habits and metabolic mechanism of methanogens [11–13]. The results indicated that the methanogens from sediments and animals were distinct, and the methanogens from freshwater and seawater were also different. Methanogens from the same sediments or animals have a high similarity, reflecting the close correlation between the ecological environment and the distribution of methanogens [14].

CH_4_ emission from lakes and ponds is a key root of atmospheric methane [15–17]. Methanogens, as the main role of methane generation, are usually characterized by the diversity or abundance [18–20]. Some scholars have studied the seasonal fluctuations of the quantity, structure, and variety of methanogens and methanotrophic bacteria in sediment from lakes, and determined that the diversity of methanotrophic bacteria was dominant by methylobacter in the deep and methylococcus in the shallows part, and organic matters was the key environmental parameters that controlled methanogens [13]. In this study, we collected samples of black carp (*Mylopharyngodon piceus*), grass carp (*Ctenopharyngodon idella*) and cultured water from the similar pond, and analyzed their methanogens community structure and diversity based on two different kinds of predatory fish intestine and their cultured water. The results will help to deepen the understanding of methanogens in the ecological functions of matter and energy circulation and transformation in the rural aquaculture pond ecosystem and provide reference information for the ecological diversity of rural aquaculture ponds and the possible impact of aquaculture on the greenhouse effect.

## Materials and methods

### Sample collection and preservation

Samples of grass carp (*Ctenopharyngodon idella*, CY), black carp (*Mylopharyngodon piceus*, QY) and farmed water (SY) were gathered from the similar lake of Loudi Fishery Science Research Institute (112°0’6" E, 27°43’47" N), Hunan Province, China. The lake has an area of 1.5 hm^2^ and a depth of 2.0 m. The sampling time on January 19, 2021 at 08:00 a.m, pH 8.10–8.56 and dissolve oxygen > 4.35 mg/L with water temperature 17.8°C. Five black carps (2627.3 ± 42.69 g) and five grass carps (1431.34 ± 33.25 g) of similar size, ailment-free symptoms were arbitrarily chosen from the fish trapped in the net and returned to laboratory along with specimen of water, and the rest were released back into the lake. The fish used in the experiment came from natural lake that had not been fed any food. At the same time, 8 sampling points were randomly selected in the lake, and 10 mL equal volume water samples were collected at places about 1.0 m below the water surface. The samples were mixed and loaded into sterile centrifuge tubes [21].

In order to prevent contaminants, the fish surface was clean with sterilized water and 70 percent ethanol sequentially prior to dissection. The content specimens of grass carp (No. CY1-CY5) and black carp (No. QY1-QY5), and water specimens (No. SY1-SY5) were gathered in the sterilized operational trays, and then put in a sterile centrifuge tube and refrigerate at -20°C for later use.

### Abundance detection of methanogens

The Tguide S96 kit was utilized to extract DNA from 15 specimens, and the universal primer MLf and MLr was use to amplify methanogenic mcrA gene. After qualified the products of PCR were identified using electrophoresis, the target fragment was recovered, and Illumina HiSeq 2500 sequenced the library. The primer, reactions system and amplification requirement are listed below. Primers were synthesized and sequenced by Beijing Biomarker Technology Co., Ltd (Beijing, China).

Amplification primer: MLr (5’-TTCATTGCRTAGTTWGGRTAGTT-3’), MLf (5’-GGTGGTGTMGGATTCACACARTAYGCWA CAGC-3’). The amplification response was achieved in the following manner: 5 μL KOD FX Neo Buffer, 0.3 μL (10 μM) comprising both forward and reverse primer, 0.2 μL KOD FX Neo, 2 μL (2 mM) of dNTPs, and 5–50 ng of DNA Template, ddH2O supplement to 10 μL. Parameters for the reaction: Denaturing at 95°C for 5 minutes, followed by 25 cycles of denaturation at 95°C for 30 seconds, annealing at 50°C for 30 seconds, and extension at 72°C for 40 seconds, with a final extension of 7 minutes at 72°C.

### Diversity analysis of methanogens

The initial data received by sequencing were filtered by quality control (Trimmomatic, V0.33) [22], and the detection and elimination of primer sequences (Cutadapt, V1.9.1) [23]. Double-terminated sequence splicing (Usearch, V10) [24] and removal of chimeras (UCHIME, V4.2) [25] obtained high-quality sequences for further investigation. Usearch software can cluster reads with 97% similarity to obtain OTU [26]. QIIME2 software (https://qiime2.org/) was employed to determine alpha and beta variety in the specimens to thoroughly examine total diversity and highlight discrepancies across specimens. Alpha diversity reflected the richness and variety of species in individual samples, containing Ace, Chao1, Simpson and Shannon. The Chao1 and Ace indices quantify species abundance, which is how many species there are. Shannon and Simpson indexes measure the variety of species and are influenced by species abundance and community evenness in sampling communities. Beta diversity examination is used to evaluate the similarity in composition of different specimens. Unweighted Pair-group Method with Arithmetic Mean (UPGMA) examine the variation between samples based on the differences in evolutionary information between different sample sequences. It can reflect whether the specimens have major microbial community variations in the evolutionary tree.

Line Discriminant Analysis Effect Size (LEfSe) [27] uses linear discriminant analysis to quantify the influence of relative number of every species on the varied effect sizes, and looked for species with substantial variations among groups.

### Statistical analysis

SPSS 24.0 statistical software (IBM Corp., Armonk, NY, USA) was use for data statistics, the testing data are represented by means ± standard deviation, and independent sample *t-test* was employed for pair comparison. *p* < 0.05 revealed statistically significant difference. The mcrA gene sequences acquired in this research has been uploaded as an attachment with the file name “raw data”.

### Ethics statement

All animal work was performed in compliance with the recommendations of the Institutional Animal Care and Use Committee of Hunan University of Chinese Medicine (NO.20171202). All writers were aware and agreed of this animal experimentation.

## Results

### Sequencing characteristics and OTU distribution of samples

**Fig 1:**
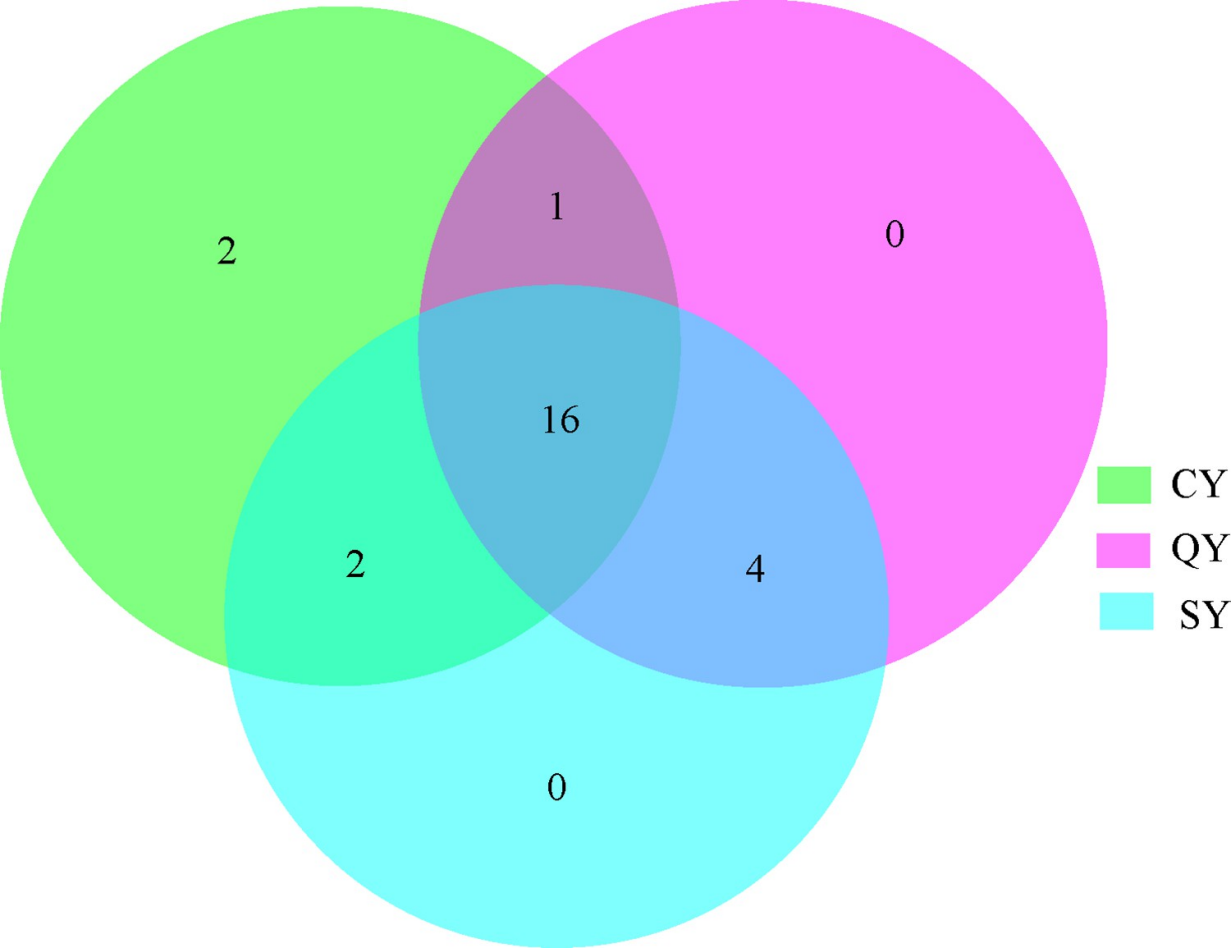


The community features of methanogens in black carp grass carp, and water specimens were examined using Illumina high-throughput sequencing technology. Approximately 1,512,019 high-quality sequences have been gathered from 15 specimens of the three groups, and the average effective sequence of each sample was 90.99%, with the average sequence length concentrated in 421–435 bp. Through quality control, filtering and chimerism removal, overall 25 OTUs were obtained based on 97% sequence similarities clustering. There were 21 OTU in CY samples. There were 21 OTU in QY samples. There were 22 OTU in SY samples. There were 16 identical OTU numbers in the three groups of samples. The outcomes revealed that there were no differences in the richness and variety of methanogens among black carp, grass carp and water samples (Fig 1).

### Comparison of alpha diversity of methanogens in black carp grass carp, and water specimens

**Fig 2:**
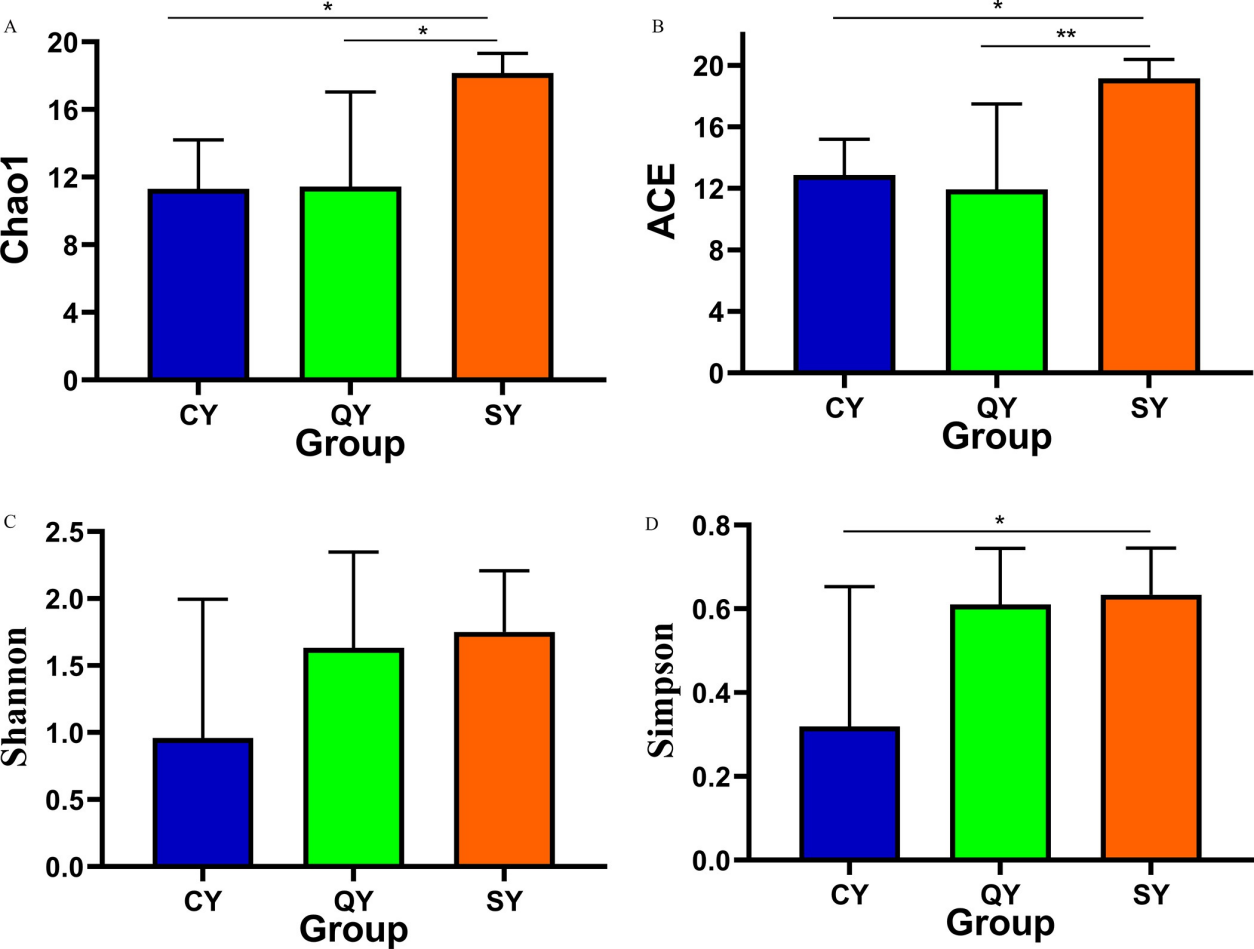


To illustrate the variety and depth of methanogens in the digestive tract of black carp, grass carp and water, QIIME2 software was used to assess the alpha diversity index of samples. In term of the perspective of richness index, Ace index and Chao1 index in water samples were the most advanced, and Ace index and Chao1 index in intestinal samples of grass and black carp were similar. From the perspective of diversity index, the Shannon and Simpson index of species in aquaculture water samples were the highest, which were similar to those in the intestinal specimen of black carp, while Shannon and Simpson index were the smallest in the digestive specimens of grass carp. The richness index in the colonel samples of black and grass carp was significantly different from that in the water specimens (*p* < 0.05 or *p* < 0.01). The diversity index in the colonel specimens of black carp and water samples was similar, and the diversity index in the colonel samples of grass carp was the lowest (Table 1, Fig 2). These outcomes designated that the richness and diversity of methanogens in water were the greatest. The richness of methanogens in the colonel tract of grass and black carp was similar, which was considerably different from that of water samples. The variety of the digestive tract samples of black carp were similar to that of water samples, but there was no significant difference.

**Table 1:** Difference analysis of alpha diversity index.

group	Chao1	Ace	Simpson	Shannon
CY	11.30±2.9069*	12.8623±2.3313*	0.3187±0.3345*	0.9594±1.0360
QY	11.44±5.6007*	11.9343±5.5446**	0.6105±0.1340	1.6332±0.7127
SY	18.15±1.1673	19.1511±1.2232	0.6339±0.1111	1.7513±0.4562

Note: CY stands for grass carp, QY stands for black carp; SY stands for water specimen; Compared with water sample *stands for *p* < 0.05,**stands for *p* < 0.01

### Characterization of methanogens in black carp, grass carp and water specimens

**Fig 3:**
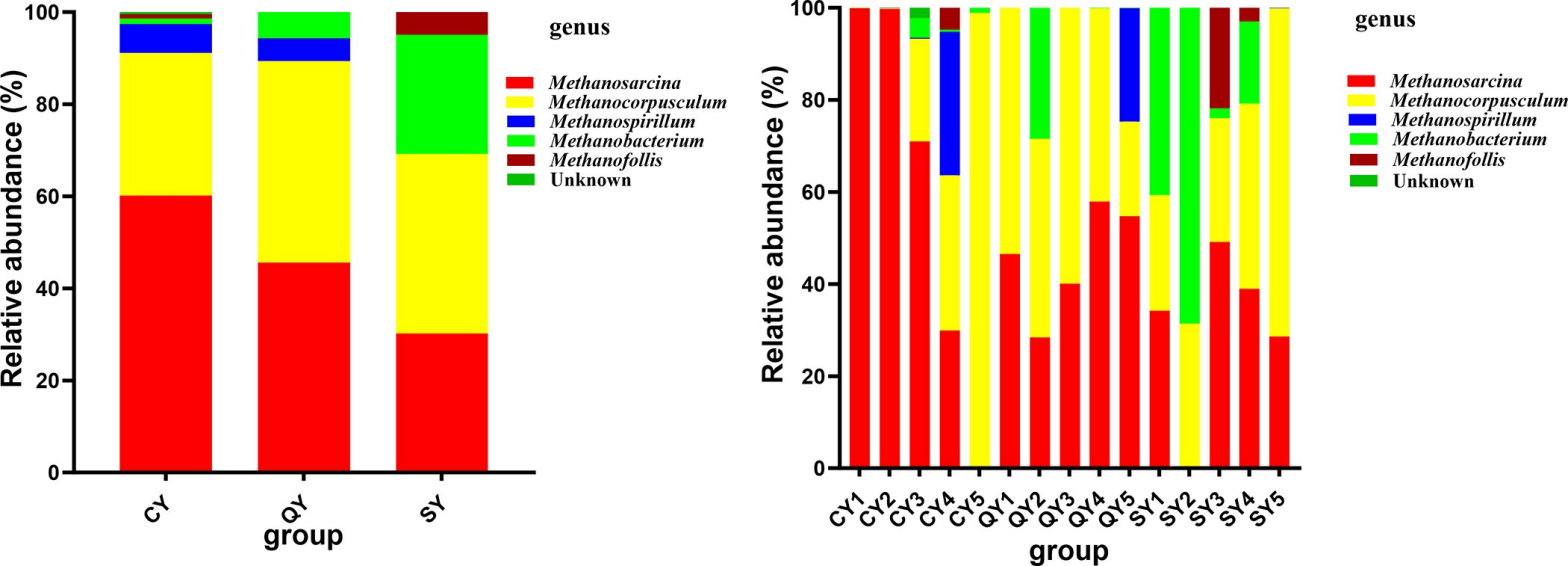


There were 3 classes, 4 orders, 5 families and 5 genera of methanogens were recognized from 15 specimens gathered from CY, QY and SY groups. The genera detected were *Methanosarcina*, *Methanocorpusculum*, *Methanospirillum*, *Methanobacterium* and *Methanofollis*. *Methanosarcina* and *Methanocorpusculum* were the dominant genera, accounting for 91.15%, 89.36% and 69.17% of CY, QY and SY, respectively (Fig 3, Table 2). Compared with SY samples, *Methanosarcina* and *Methanospirillum* increased in CY and QY samples to varying degrees. *Methanobacterium* and *Methanofollis* have decreased in CY and QY samples to varying degrees. *Methanocorpusculum* increased in QY samples but decreased in CY samples. There were no significant differences among all genera.

**Table 2:** Relative abundance of methanogens at genus level.

genus	CY	QY	SY
*Methanosarcina*	0.6014±0.4409	0.4555±0.1186	0.3020±0.1841
*Methanocorpusculum*	0.3101±0.4052	0.4380±0.1498	0.3898±0.1904
*Methanospirillum*	0.0628±0.1391	0.0493±0.1101	0.0002±0.0001
*Methanobacterium*	0.0117±0.0180	0.0571±0.1271	0.2584±0.2888
*Methanofollis*	0.0095±0.0212	0.0001±0.0000	0.0497±0.0951

Note: CY stands for grass carp, QY stands for black carp; SY stands for water specimen; Compared with water sample *stands for *p* < 0.05,**stands for *p* < 0.01

**Fig 4:**
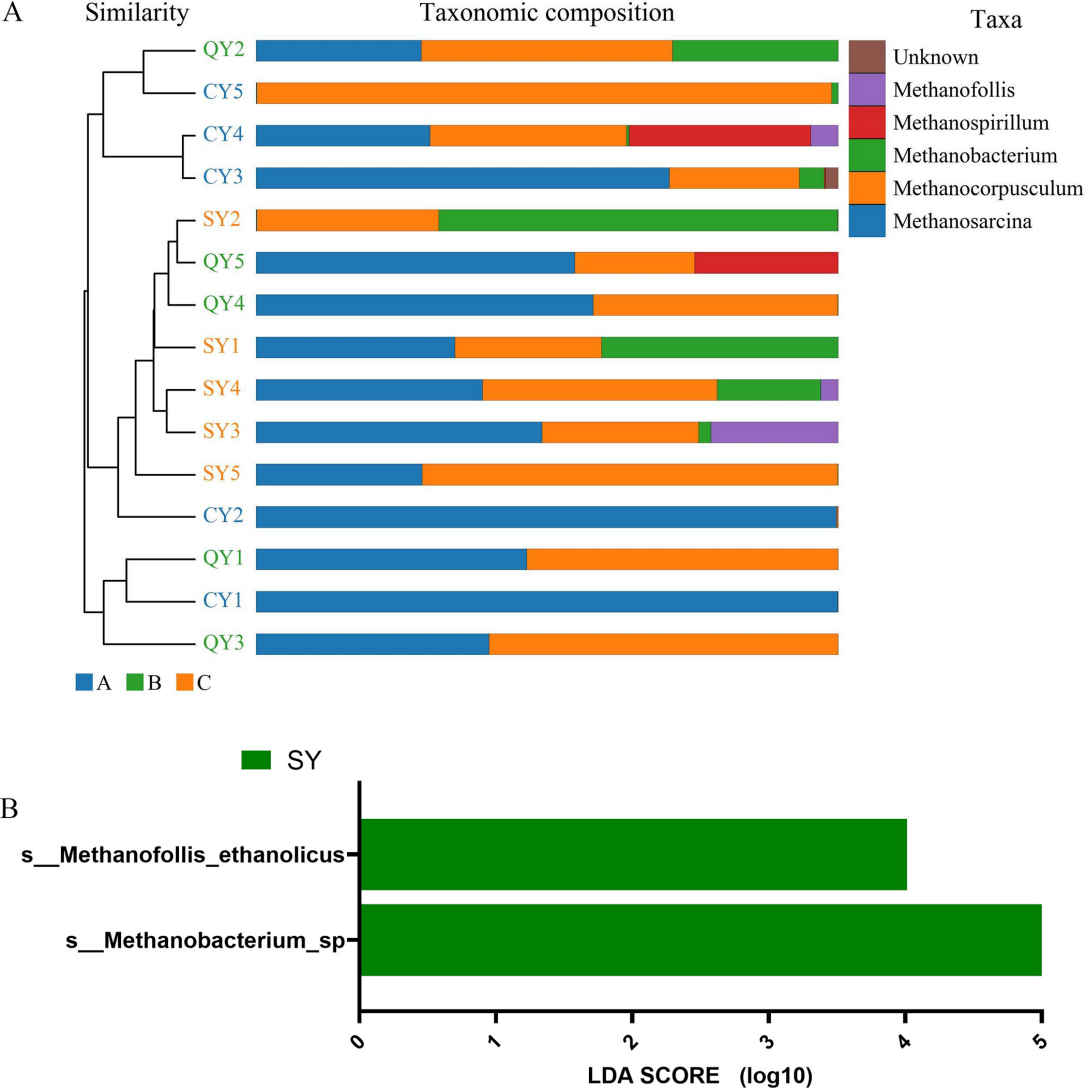


Weighted Unifrac distance matrix was utilized to construct a cluster examination of fifteen samples known as methanobacteria by means of the unweighted pair-group method with UPGMA. The outcomes revealed that the resemblance of species composition among each group was relatively high (Fig 4). Based on the LefSe analysis findings, *Methanobacterium*_sp and *Methanofollis*_ethanolicus are markers with statistical differences in SY samples (Fig 4).

## Discussion

Understanding variations in the abundance and variety of microbial colonies is a necessary condition for evaluating the role of microorganisms in the environment [13]. Methanogens, as common microorganisms, are extensively dispersed in numerous environments, containing soil [28, 29], water sediments [30, 31] and animal digestive tracts [32, 33]. Ponds and lakes are important natural emission sources of methane, and methane generation is closely related to the methanogens community. At present, three main metabolic pathways have been described for methane production, namely hydrogenotrophic (change H_2_ plus CO_2_ into CH_4_), aceticlastic (change acetate into CO_2_ and CH_4_) and methylotrophic (generate CH_4_ by methanol, methylamine, dimethylamine and other mechanisms), which involve the diversity of methanogens [34–36]. In freshwater sediments, methane production is regulated by different environmental factors, such as hypoxia [37], quality and amount of organic matter [18, 38], temperature [39], etc. Temperature variation is probably to be one of the factors affecting CH_4_ production capacity in the shallowest areas of deep lakes or shallow lakes [40]. In a certain range, the increase in temperature has an obvious promotion effect about the metabolic capability of microorganisms, which is beneficial to improve the rate of gas production. In addition, the production capacity of CH_4_ is closely related to the community abundance of fermentation microorganisms [41]. Therefore, it is helpful to clarify the relationship between aquaculture and the greenhouse effect to study the community characteristics of methanogens in aquaculture water and digestive tracts of aquatic animals.

In this investigation, High-throughput sequencing technologies were used to methanogens in water samples and aquatic animal intestines. The results revealed that a total of 5 genera were identified from methanogens, among which *Methanosarcina*, *Methanocorpusculum* and *Methanobacterium* were the 3 genera with the greatest relative abundance. *Methanosarcina* is hydrogen and acetic acid mixotrophic methanogens, *Methanocorpusculum* and *Methanobacterium* are hydrogenotrophic methanogens [41]. The results showed that CH_4_ was produced by H_2_ reduction of CO_2_ and acetic acid degradation, and mainly by hydrogen reduction of CO_2_. In the SY sample, *Methanocorpusculum*, *Methanosarcina*, and *Methanobacterium* were the dominant bacteria, which is constant with the characteristics of methanogenic bacteria community in wetlands [42, 43]. In QY and CY samples, *Methanosarcina* and *Methanocorpusculum* were the dominant bacteria genera. In addition, *Methanosarcina* has the highest abundance in CY and *Methanocorpusculum* has the highest abundance in QY, which might be link the eating habits of black and grass carp.

The grass carp is herbivorous freshwater fish that feeds on the stems and leaves of aquatic plants, and its food riched in cellulose and polysaccharides. Black carp are carnivorous fish that live in freshwater, which feeds on snails, clams and other mollusks, and its food is rich in protein and fat. The intestinal bacteria of grass and black carp are mainly Firmicutes (69% vs 37.5%), Proteobacteria (6.9% vs 37.5%) and Actinobacteria (6.9% vs 16.7%), which are highly similar to the bacterial community in cultured water [44–46]. Firmicutes, Proteobacteria and Actinomycetes belong to hydrolytic fermentation bacteria [47]. Among them, *Clostridium* in Firmicutes is a typical cellulose-decomposing bacteria with the function of fermenting monosaccharides to produce organic acids, while *Streptococcus* in Firmicutes is a typical protein-decomposing bacteria [48, 49]. Vibrio in Proteobacteria is the dominant lipopolysis bacteria [50]. Moreover, the colonel tract of grass carp is rich in amylase and cellulose [51], and the digestive tract of black carp is rich in protease and lipase [52]. These facts indicate that metabolic matrix of methanogens in ponds and lakes mainly comes from hydrolytic fermentative bacteria, and methanogens can effectively use the H_2_ and CO_2_ generated by these hydrolytic fermentative bacteria to produce CH_4_, which also fully demonstrates that ponds and lakes are important natural emission sources of methane, and are dominated by hydrogenotrophic methanogens.

In conclusion, we revealed the community structure and richness characteristics of methanogens in the colonel tract of black and grass carp and water samples for the first time and clarified the relationship between intestinal methanogens and aquaculture and the greenhouse effect. These results will provide a reference for the relationship between intestinal methanogens and aquaculture and the greenhouse effect.

## Supporting information

S1 Raw data (ZIP)
